# Supplementary material for: Analysis of genetic diversity and population structure of Magnaporthe grisea, the causal agent of foxtail millet blast using microsatellites
Source: PeerJ. 2023 Oct 31;11:e16258. doi: 10.7717/peerj.16258 (PMC10624167; doi:10.7717/peerj.16258)
Supplement: Supplemental Information 2 — Supplementary Table [file peerj-11-16258-s002.doc]

**TABLE S1. Details of SSR markers used for the genetic analysis of *M. grisea***

| **S. No** | **Marker** | **Primer sequence**  **(5’-3’)** | **Source** | **SSR type** | **T( ̊ C)** | **Expected amplicon size** |
| --- | --- | --- | --- | --- | --- | --- |
| 1. | Pyrms 7 | GCAAATAACATAGGAAAACG | Full BAC (70-15) | (CT/GA)_29_ | 53 ̊ C | 123-179 |
|  | Pyrms 8 | AGAAAGAGACAAAACACTGG |  |  |  |  |
| 2. | Pyrms 15 | TTCTTCCATTTCTCTCGTCTTC | EST (P12) | (CT/GA)_20_ | 53 ̊ C | 151-200 |
|  | Pyrms 16 | CGATTGTGGGGTATGTGATAG |  |  |  |  |
| 3. | Pyrms 37 | ACCCTACCCCCACTCATTTC | BAC end (70-15) | (CA/GT)_6_ +  (CT/GA)_12_ | 53 ̊ C | 196-205 |
|  | Pyrms 38 | AGGATCAGCCAATGCCAAGT |  |  |  |  |
| 4. | Pyrms 39 | CGCATACAGGAAAGCCAAGA | EST (Guy11) | CA/GT_19_ | 53 ̊ C | 500-530 |
|  | Pyrms 40 | CTGACGAGGGACTCCTGTGT |  |  |  |  |
| 5. | Pyrms 41 | AACGTGACAATGTGAGCAGC | BAC end (70-15) | CT/GA_16_ | 55 ̊ C | 119-193 |
|  | Pyrms 42 | GCCATGTTCTAAGGTGCTGAG |  |  |  |  |
| 6. | Pyrms 43 | TCAGTAGGCTTGGAATTGAAAAA | BAC end (70-15) | TA /AT_12_ | 55 ̊ C | 190-233 |
|  | Pyrms 44 | CTTGATTGGTGGTGGTGTTG |  |  |  |  |
| 7. | Pyrms 45 | CCACTTTATAGCCCACCCAGT | BAC end (70-15) | TA/AT_11_ | 55 ̊ C | 203-223 |
|  | Pyrms 46 | CTCTTTTCTCGCAGGAGGTG |  |  |  |  |
| 8. | Pyrms 47 | TCACATTTGCTTGCTGGAGT | BAC end (70-15) | TA/AT_15_ | 55 ̊ C | 182-206 |
|  | Pyrms 48 | AGACAGGGTTGACGGCTAAA |  |  |  |  |
| 9. | Pyrms 59 | TTCTCAGTAGGCTTGGAATTGA | BAC end (70-15) | TA/AT_12_ | 55 ̊ C | 183-212 |
|  | Pyrms 60 | CTTGATTGGTGGTGGTGTTG |  |  |  |  |
| 10. | Pyrms 61 | GAGGCAACTTGGCATCTACC | BAC end (70-15) | GA/CT_9_ | 55 ̊ C | 230-281 |
|  | Pyrms 62 | TGGATTACAGAGGCGTTCG |  |  |  |  |
| 11. | Pyrms 63 | TTGGGATCTTCGGTAAGACG | BAC end (70-15) | CT/GA_15_ | 55 ̊ C | 163-183 |
|  | Pyrms 64 | GCCGACAAGACACTGAATGA |  |  |  |  |
| 12. | Pyrms 67 | AGCAAGCAGGAGATGCAGAC | SSR library (Guy11) | CA/GT_17_ | 55 ̊ C | 191-233 |
|  | Pyrms 68 | GTTTGGCTGGCAAGACAGTT |  |  |  |  |
| 13. | Pyrms 77 | GAAGTATTGCACACAAACAC | SSR library (Guy11) | CA/GT_24_ | 55 ̊ C | 162-240 |
|  | Pyrms 78 | GCTTTCGGCAAGCCTAATC |  |  |  |  |
| 14. | Pyrms 81 | CCTTGTTTTCCCCCTGTGTA | BAC end (70-15) | ACT/TGA_12_ | 55 ̊ C | 150-190 |
|  | Pyrms 82 | TAGCCAAATGCCCATTATCC |  |  |  |  |
| 15. | Pyrms 83 | GTCTGCCTCGACTCCTTCAC | BAC end (70-15) | TCA/AGT_13_ | 55 ̊ C | 168-189 |
|  | Pyrms 84 | AGCCCAAAAACAGAAAGCAA |  |  |  |  |
| 16. | Pyrms 87 | AGACTTGTTACTCGGGTCTTGA | BAC end (70-15) | TGC/ACG_12_ | 53 ̊ C | 180-195 |
|  | Pyrms 88 | CCAGATGTCACTCCCCTGTA |  |  |  |  |
| 17. | Pyrms 93 | CCTCGACTCCTTCACCAAAA | Est (70-15) | ATC/TAC_12.5_ | 55 ̊ C | 214-235 |
|  | Pyrms 94 | CGGAGAGCTCAGGAAGAGG |  |  |  |  |
| 18. | Pyrms 99 | CACCACTTTATGGCGCAGT | BAC end (70-15) | ACC/TGG_20_ | 53 ̊ C | 192-225 |
|  | Pyrms 100 | ACCTAGGTAGGTATACATGTTGTT |  |  |  |  |
| 19. | Pyrms 101 | CTGCGTTCAACATGCCTCTA | SSR library (Guy11) | TG/AC_25_ | 53 ̊ C | 177-205 |
|  | Pyrms 102 | CTTAGATCTGCGGTATGAGCA |  |  |  |  |
| 20. | Pyrms 107 | GCAGCAAGCAGCAATATCAG | SSR library (Guy11) | GA/CT_10_ | 53 ̊ C | 344-384 |
|  | Pyrms 108 | GTGGATATCGAAGGCCAAGG |  |  |  |  |
| 21. | Pyrms 109 | TACAGTGGGAGGGCAAAGAG | SSR library (Guy11) | TG/AC_12_ | 55 ̊ C | 192-225 |
|  | Pyrms 110 | CCAGATCGAGAAGGGGGTAT |  |  |  |  |
| 22. | Pyrms 125 | CTCTCCGGCCAAGATTGA | Full BAC (70-15) | CAA/GTT_32_ | 55 ̊ C | 133-190 |
|  | Pyrms 126 | GGTTGTTGGGAGAAAGAACG |  |  |  |  |
| 23. | Pyrms 233 | TGAGATGGACCGCATGATTA | Genome sequence | CAG/ GTC_10_ | 55 ̊ C | 251-269 |
|  | Pyrms 234 | TTGATGGCAGAGACATGTAGC |  |  |  |  |
| 24. | Pyrms 319 | TAAGACCACTGGCGGAATCT | Genome sequence | CAA/GTT_6_ | 55 ̊ C | 280-295 |
|  | Pyrms 320 | GGCTTTGTCTGGTTGTACGG |  |  |  |  |
| 25. | Pyrms 427 | CTGTCACCACAACCAAGACG | Genome sequence | AT/TA_16_ | 55 ̊ C | 200-220 |
|  | Pyrms 428 | TTGCCCTGATTTGTCAGTCA |  |  |  |  |
| 26. | Pyrms 453 | GACCCTCGAGAAATGATGGA | Genome sequence | GAA/CTT_6_ | 55 ̊ C | 293-297 |
|  | Pyrms 454 | GTCAAAGAGCTCCCCATCTG |  |  |  |  |
| 27. | Pyrms 533 | CTTATCGGGAGGTGCAGAAG | Genome sequence | TCTAGT/AGATCA_3_ | 55 ̊ C | 255-295 |
|  | Pyrms 534 | GACGGCATACTGCATACGTG |  |  |  |  |
| 28. | Pyrms 607 | CCCAAGCTCCATAATAGGCTAC | Genome sequence | GCA/CGT_13_ | 55 ̊ C | 270-309 |
|  | Pyrms 608 | TCCGAGACTCTTTGGATAGCAC |  |  |  |  |
| 29. | Pyrms 657 | ATCAGTCGAACCCACAAAGC | Genome sequence | CA/GT_12_ | 55 ̊ C | 155-200 |
|  | Pyrms 658 | ATGTGTGGACGAACCAGTCC |  |  |  |  |

Note: Primer pairs 1-22 were referred from Kaye et al. (2003) and 23-29 from Adreit et al. (2007)

**TABLE S2. Evanno table for structure analysis**

| **K** | **Reps** | **Mean LnP(K)** | **Stdev LnP(K)** | **Ln'(K)** | **|Ln''(K)|** | **Delta K** |
| --- | --- | --- | --- | --- | --- | --- |
| 1 | 5 | -3072.56 | 1.0383 | NA | NA | NA |
| 2 | 5 | -2657.54 | 0.9737 | 415.02 | 214.04 | 219.83193 |
| 3 | 5 | -2456.56 | 2.5793 | 200.98 | 56.58 | 21.935836 |
| 4 | 5 | -2312.16 | 14.0047 | 144.4 | 22.74 | 1.623735 |
| 5 | 5 | -2190.5 | 24.5833 | 121.66 | 2.4 | 0.097627 |
| 6 | 5 | -2066.44 | 25.0466 | 124.06 | 24.54 | 0.979773 |
| 7 | 5 | -1966.92 | 20.5206 | 99.52 | 4.86 | 0.236835 |
| 8 | 5 | -1862.54 | 28.2093 | 104.38 | 16.04 | 0.568607 |
| 9 | 5 | -1774.2 | 31.6267 | 88.34 | 9.56 | 0.302277 |
| 10 | 5 | -1676.3 | 19.9252 | 97.9 | NA | NA |

**TABLE S3. Summary of analysis of molecular variance (AMOVA) for hierarchical clustering approach**

| **Source** | **Degree of freedom** | **Sum of square** | **Mean squares** | **Estimated variance** | **Percentage of variation** |
| --- | --- | --- | --- | --- | --- |
| Among Pops | 2 | 89.192 | 44.596 | 1.697 | 20% |
| Among Indiv | 29 | 386.074 | 13.313 | 6.602 | 79% |
| Within Indiv | 32 | 3.500 | 0.109 | 0.109 | 1% |
| Total | 63 | 478.766 | 58.018 | 8.408 | 100% |
| **F-Statistics** | **Value** | **P** |  |  |  |
| F_st_ | 0.202 | 0.001 |  |  |  |
| F_is_ | 0.984 | 0.001 |  |  |  |
| F_it_ | 0.987 | 0.001 |  |  |  |

**TABLE S4a. Percentage of variation explained by the first 3 axes**

| **Axis** | **1** | **2** | **3** |
| --- | --- | --- | --- |
| Variation of individual axis (%) | 18.03 | 9.13 | 6.85 |
| Cumulative variation (%) | 18.03 | 27.16 | 34.00 |

**TABLE S4b. Nei genetic diversity Percentage of variation explained by the first 3 axes**

| **Axis** | **1** | **2** | **3** |
| --- | --- | --- | --- |
| Variation of individual axis (%) | 57.84 | 42.16 | 0.00 |
| Cumulative variation (%) | 57.84 | 100.00 | 100.00 |
