# Supplementary figures and images for: Analysis of genetic diversity and population structure of Magnaporthe grisea, the causal agent of foxtail millet blast using microsatellites
Source: PeerJ. 2023 Oct 31;11:e16258. doi: 10.7717/peerj.16258 (PMC10624167; doi:10.7717/peerj.16258)

**FIGURE S1**

**
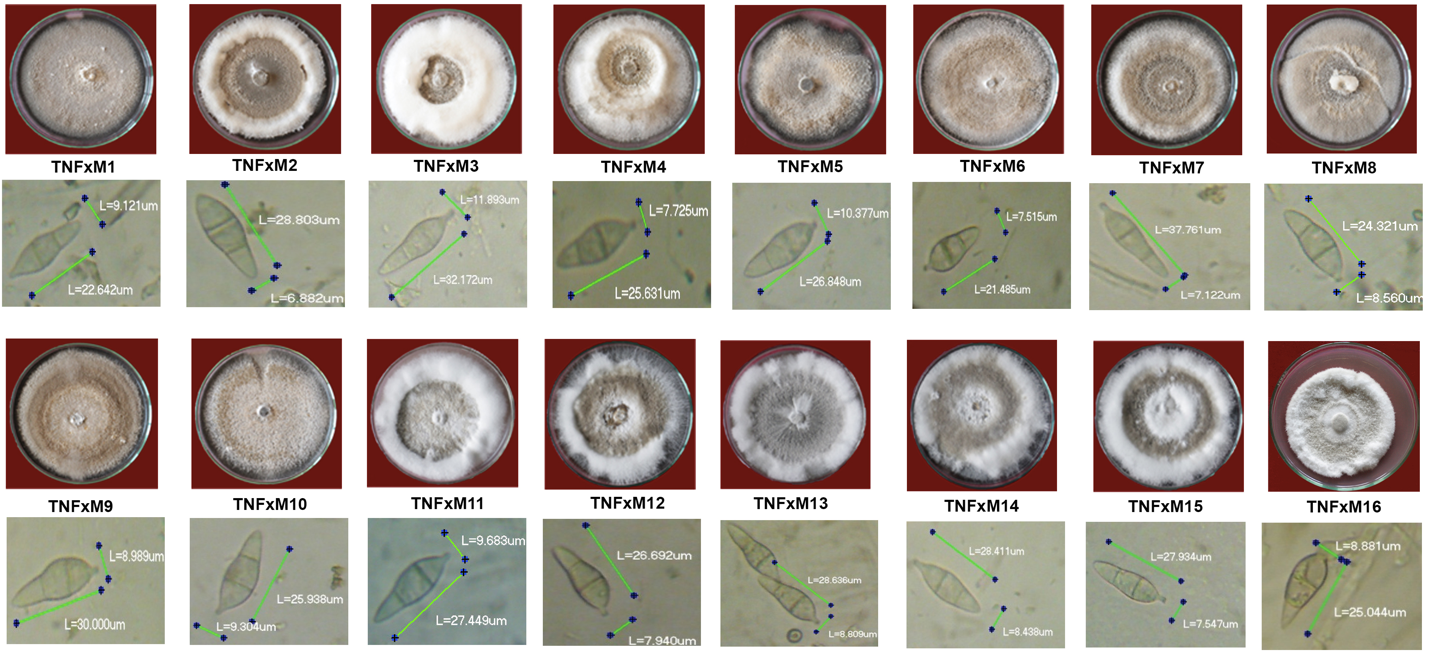
**

**
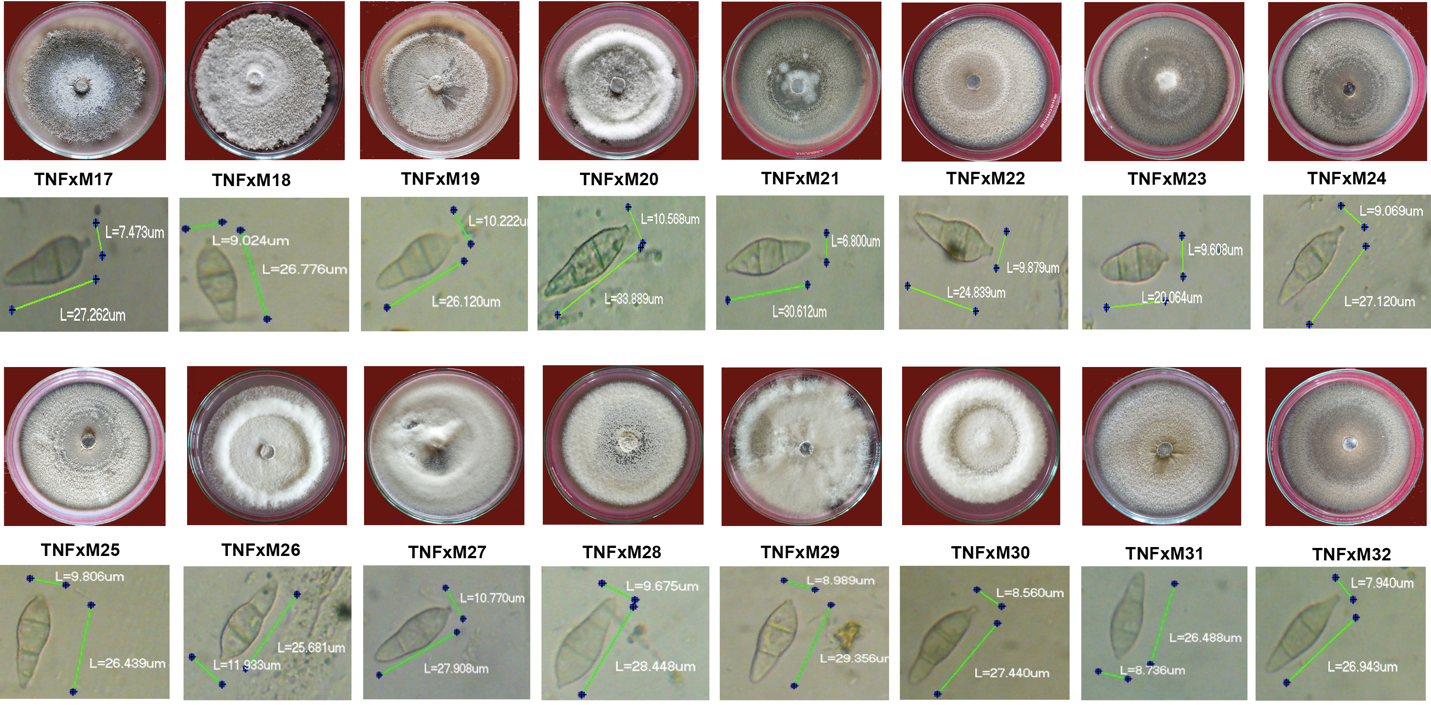
**

**FIGURE S2**

**
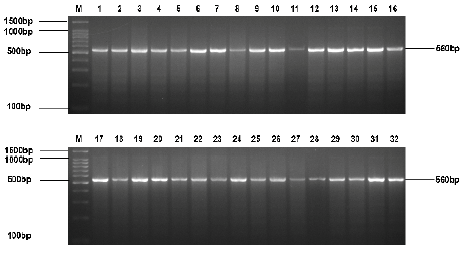
**

**FIGURE S3**

**(a)**


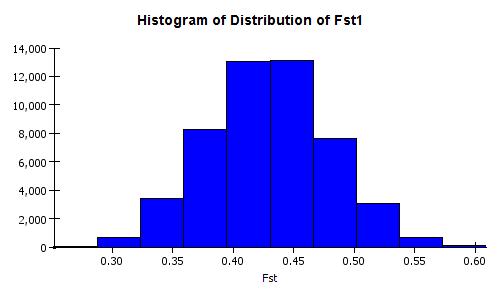


**(b)**


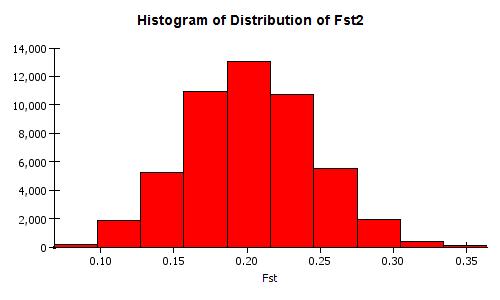


**(c)**


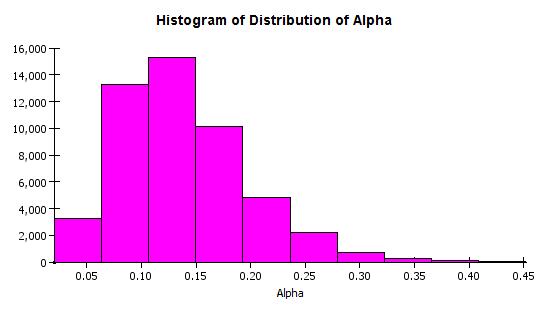

Supplement: Supplemental Information 3 [file peerj-11-16258-s003.docx]
